# Superior absorption and retention properties of foam-film silver dressing versus other commercially available silver dressing

**DOI:** 10.1186/s40824-016-0069-z

**Published:** 2016-08-06

**Authors:** Seung Moon Lee, Il Kyu Park, Yong Soo Kim, Hyun Jung Kim, Hanlim Moon, Stefan Mueller, Harsha Arumugam, Young-IL Jeong

**Affiliations:** 1 Genewel Co. Ltd., Gyeonggi-do, Korea; 2 Mundipharma Pte. Ltd., Singapore, 018961 Singapore; 3 Biomedical Research Institute, Pusan National University Hospital, 179 Gudeok-ro, Seo-gu, Busan, 602-739 Republic of Korea

**Keywords:** Wound healing, Silver-containing dressing, Polyurethane foam dressing, Porosity, Absorption/retention

## Abstract

**Background:**

The aim of this study is to investigate the physicochemical and structural properties of Medifoam®Silver and to compare with other commercially available silver-containing polyurethane (PU) foam dressing in vitro.

**Methods:**

Surface and cross-section of four polyurethane foam dressings were assessed with field-emission scanning electron microscope. Thickness, density, tensile strength, elongation, absorption rate, absorption/retention capacity and water-vapor transmission (WVT) were measured to compare physical properties of various dressing materials.

**Results:**

Among four tested dressings, Medifoam®Silver has relatively uniform and smallest pore size in both surface and cross-section. In comparison of absorption properties with other dressing materials, Medifoam®Silver has rapid absorption rate, good absorption/retention capacity and good WVT value.

**Conclusions:**

The data further suggests that Medifoam®Silver is a promising candidate for wound healing management.

## Background

The aim of wound dressings is to promote tissue regeneration and ultimately to speed up wound healing, to reduce infections and minimize pain [1]. The benefits of an optimal moist environment at the wound interface in terms of facilitating wound closure and spending up the healing process are well described [2]. Wet wound dressings may provide optimal conditions not only for proliferation of healthy cells but also for bacterial growth [3]. Furthermore, the absorption/retention performance of wet wound dressing material drives absorption of exudate and plays an important role in moisturized wound environment. Subsequently, the balanced moist environment at the wound interface is known to facilitate closure and fast healing process [4].

Wound infection commonly occurs from inappropriate cleansing, debridement, dressing techniques as well as exposure to contaminated sources, and represents a major barrier to wound healing [5]. Due to potential bacterial resistance, topical use of antibiotics for non-infected wound is discouraged but still it is very commonly used [6]. Topical silver creams and solutions have been extensively used for wound healing since they offer a broad spectrum of antimicrobial activity, less development of resistance, fewer adverse reactions, and a low risk of systemic toxicity [7, 8]. However, these agents normally require frequent application, care-intensive application/removal, and cause painful reaction in patients [8–10]. Since silver-compounds such as silver nitrate and silver sulfadiazine is known to have antibacterial activity, it has been widely used in wound healing treatment as a silver-containing dressings or topical creams [11, 12]. Many reports describe that silver-based dressings are helpful to overcome bacterial infections, to promote environment for granulation/re-epithelialization in wound area, reduce adverse effects and to facilitate wound healing [2, 3, 10, 11].

Modern silver dressings should maintain moisture balance by absorbing excess exudate and preventing the wound drying out, while conferring antibacterial protection in wounds at risk of infection [2, 4]. The physical property of foam dressing should be maintained when it is coated with silver. In our previous report, we showed that our wound dressing, Medifoam® N, has excellent physicochemical properties when compared with other dressing materials [13]. Furthermore, we also showed that appropriate physicochemical properties of wound dressing material such as porous structure, thickness/density, tensile strength and moisture-vapor transmission rate contributed to improve performance of wound dressing material. Since silver-compound has been demonstrated in the protection of bacterial contamination and then facilitation of re-epithelialization in the wound area, we developed silver-containing wound dressing (Medifoam®Silver), it may contribute to improve efficacy of wound dressing material against contamination circumstances and to facilitate wound healing. Furthermore, excellent physicochemical proeprties of our wound dressing material may also contribute to improve efficiency of wound healing. For example, Yunoki et al., reported that physicochemical properties of wound dressing such as release of silver was contributed to antibacterial activity of wound dressing material [14]. Furthermore, other researchers also investigated physicochemical properties and antimicrobial efficiency [15, 16].

This study was conducted to compare physicochemical properties including pore size, absorption and retention capacity, and in vitro antibacterial activities of Medifoam®Silver to those of other marekted silver-dressings.

## Methods

### Materials

Silver impregnated dressings such as Medifoam®Silver (Genewel Co., Ltd, South Korea), AAG (Allevyn® AG (Smith & Nephew Co. TX, USA)). MAG (Mepilex® AG (Mölnlycke Health Care Co. Sweden)) and PS (Polymem® Silver (Ferris Manufacturing Co. Illinois, USA)) were obtained or purchased from each respective company. Phosphate-buffered saline (PBS, pH 7.4, 0.01 M, with pigment (SCU656, 0.1 %, DaeBo Co. ltd, Gyeonggi-do, Korea) was purchased from Gibco (NY, USA). All organic solvents were used as HPLC grade without further purification.

### Thickness and density measurements

Thickness was measured using MDH micrometer high accuracy sub-micron digimatic micrometer (CD-15CPX, Mitutoyo Co. Ltd., Kawasaki, Japan) repeatedly at least 10 times with different samples. The value of thickness was described in mm as mean ± standard deviation.

Density was calculated from measurement of (weight, width, length and thickness) of the foam dressing materials and described in g/cm^3^.

$$ Density\ \left(\frac{g}{c{m}^3}\right)=\frac{weight}{width* length* thickness} $$

Density measurement was also repeated at least 10 times with different samples (Mean ± standard deviation).

### Morphology assessment

To assess morphology of foam dressing, field-emission scanning electron microscope (FE-SEM, S-4800, Hitachi, Tokyo, Japan) was employed. Each foam dressing was cut into uniform size and then coated by platinum coater. The prepared specimens were assessed at 25 kV. In the morphology analysis, the pore size and pore size uniformity of each dressing were measured and compared. Pore size was measured directly from the photo with a ruler. At least, more than three photos were taken from different positions and used to analyze pore size measurement.

### Tensile strength and elongation

Material test machine (3343Q9831, Instron Co., MA, USA) was used for measuring tensile strength. Foam dressings were carefully clipped out of Dog-bone (6 mm × 105 mm) using a wood-molded blade with maintenance of the surface of foam dressing unscathed. Clipped foam dressing was installed into the test machine symmetrically across the cross-section of the grip of the instrument. Foam dressings were pulled out (speed: 300 mm/min) at a snap and the value was calculated in the following equation: [Tensile Strength (kgf/cm^2^) = MAX Failure Strength (kgf)/Cross-section of Specimen (mm^2^)]. Elongation was calculated in the following equation:

$$ Elongation\ \left(\%\right)=\left(\frac{D2-D1}{D1}\right)*100 $$

D1: Initial Inter-clip Distance. D2: Inter-clip Distance at Snap.

### Absorption rate

Absorption rate was measured as follows: After dressing materials were placed onto flat surfaces, phosphate-buffered saline (PBS, pH 7.4, 0.01 M) solution was pipetted onto the wound contact layer at the height of 1.0 ± 0.5 cm. The absorption rate of PBS was measured using stopwatch, repeatedly, minimum five times. The values were expressed as mean ± standard deviation.

### Absorption and retention capacity

Absorption and retention capacity was carried out in accordance with BS EN 13726–1:20022. Five samples of foam dressings measuring 5 × 5 cm were prepared to measure absorption capacity. The baseline weight (W1) and thickness (T) of each sample was recorded. Distilled water (37.0 ± 1 °C) equal to 40 times of the weight of the sample was added to each experimental sample. Temperature of distilled water was constantly controlled with Pyrostat (OF-21E, Jeio Tech, Daejeon, Korea) at 37 °C for 30 min. After 30 min, dressings were suspended and then weighed for 30 s (W2). The absorption rate was calculated with following formula:

$$ Absorption\ \left(\frac{g}{c{m}^2}\right) = \left(\frac{W2-W1}{Initial\  area\  of\  dressing\ \left(c{m}^2\right)}\right) $$

The absorption and retention capacity was measured. Initial weight (A) and thickness (B) of dressings were measured to similar with absorption study. Once absorption rate was measured, each sample was pressed by a 5 kg weight (111209, Jongro industrial Co., Ltd., Seoul, Korea) for 20 s and then weighed again (W3). Then, the retention weight (D) was calculated with following formula: Retention capacity (g/cm^2^) = W3 – W1/initial specimen area (cm^2^). The mean absorption and retention of the samples of each product were calculated.

### Water-vapor transmission (WVT) measurement

The WVT value of dressing materials was measured as follows: Aluminum cups with diameter of 62 mm and absorptive area of 0.0028 m^2^ were preheated in a dry oven (100 °C) and placed with CaCl_2_ (20 g). Each dressing was tied onto the top of the aluminum cup. Ten glass bottles were used for each dressing. The specimen was set to face CaCl_2_ in the aluminum cup and the dead center of the cup to create a concentric ring. To seal the edges, preheated paraffin solution in a dry oven (100 °C) was poured along the edge of the absorptive cup (beaker) and then paraffin debris was removed thereafter. The joint aluminum cups containing CaCl_2_ and covering dressing was weighed for baseline (W_1_) and 24 h after it was placed into the thermo-hygrostat (37 °C, 75 % humidity) (W_2_). The MVTR of the samples were calculated as follows:

$$ WVT\ \left(\frac{g}{c{m}^2}* day\right) = \left(\frac{W2-W1}{B* day}\right) $$

- W_1_ : initial weight of the sample before put into the thermo-hygrostat.
- W_2_ : weight of the sample in thermo-hygrostat after 24 h.
- B : absorptive area (0.0028 m^2^).

### Antibacterial activity

The antimicrobial activity of the foam dressings was compared against Staphylococcus aureus and Pseudomonas aeruginosa obtained from the American Type Culture Collection (ATCC, Manassas, VA, USA); ATCC 6538 and ATCC 10145, respectively. To evaluate antimicrobial activity, the dynamic shake flask test was used. Briefly, 50 ml of culture inoculum containing approximately 1×10^6^ CFU/ml were added to a sterile 250 ml screw-cap Erlenmeyer flask (Triforestlabware, CA, USA). Bacterial concentration of solution was determined at the “0” time by performing serial dilutions and standard plate count techniques from the “inoculum only” sample flask. Each dressing (1 cm × 1 cm) was placed in the individual flask and incubated under dynamic conditions at 32 °C. Following incubation, at different time intervals (1 min, 5 min, 10 min, 20 min, 30 min, and 1 h), the dressings were removed and the resulting suspensions were incubated at 35 °C for 24 h in Petri dishes (BD(Becton Dickinson), CA, USA). The numbers of colonies (CFU/ml) for the dressing-containing flask (A) and for the “inoculum only” flask (B) were counted. The reduction of microorganisms (%) for each dressing was calculated using the following formula;

$$ \mathrm{L}\mathrm{o}{\mathrm{g}}_{10}\kern0.5em \mathrm{reduction}\ \left(\mathrm{L}\mathrm{R}\right) = \mathrm{l}\mathrm{o}{\mathrm{g}}_{10}\left(\mathrm{B}\right)\ \hbox{-}\ \mathrm{l}\mathrm{o}{\mathrm{g}}_{10}\left(\mathrm{A}\right) $$

$$ \mathrm{Percent}\ \mathrm{reduction}\ \left(\%\right) = 100 \times \left(1\ \hbox{-}\ 1{0}^{\hbox{-} \mathrm{L}\mathrm{R}}\right) $$

### Statistical analysis

All experimental data were expressed as mean ± standard deviation (S.D.). Statistical alaysis was evaluated by Student’s *t* test using Sigmaplot program (Sigmaplot version 11.2, Systat software, Inc., CA, USA) and *p* value < 0.05 was considered as statistically significant.

## Results

### Physico-chemical properties of foam dressings

**Fig. 1:**
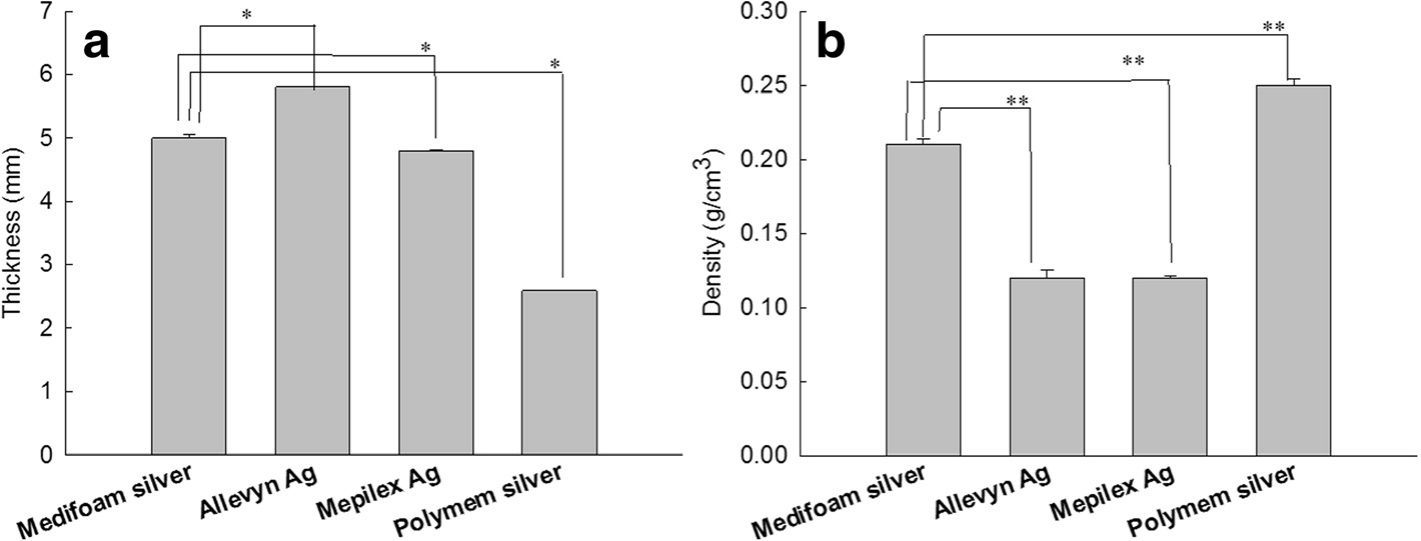
Thickness (**a**), density (**b**) of foam dressing. *,** : *p* < 0.001

**Fig. 2:**
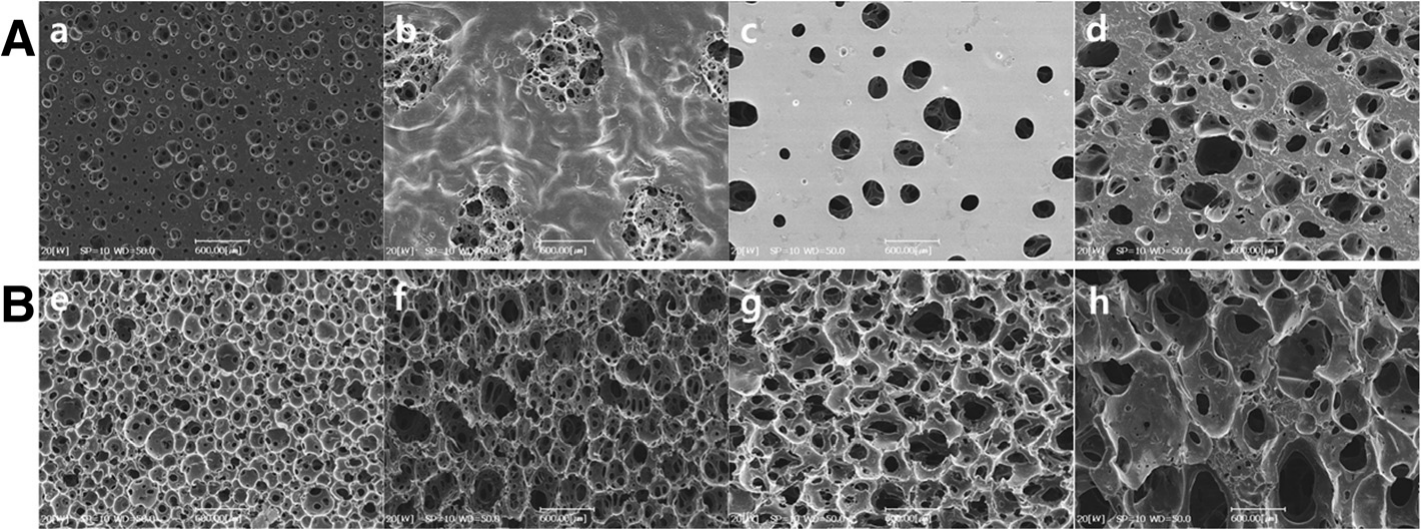
Morphological assessment of foam dressing by FE-SEM. **A** Surface (wound contact layer): (**a**) Medifoam silver, (**b**) Allevyn Ag, (**c**) Mepilex Ag, and (**d**) Polymem silver. **B** Cross-section: (**e**) Medifoam silver, (**f**) Allevyn Ag, (**g**) Mepilex Ag, and (**h**) Polymem silver

In this study, we compared the physical and structural properties of Medifoam®Silver, with several other commercially available dressings. Medifoam®Silver features an absorptive SSD-containing polyurethane foam layer between a semi-permeable absorptive polyurethane film wound contact layer and an outer layer of breathable film. Since properties of the dressing such as thickness, density, and morphology have primary importance in wound healing potential, those were evaluated as shown in Figs. 1, 2 and Table 1. As shown in Fig. 1(a), thickness of Medifoam®silver was 5.05 mm. Allevyn® Ag and Polymem®silver has largest and smallest thickness, respectively. Figure 1(b) shows density of foam dressings. Medifoam®silver and Polymem®silver showed higher density than others.

**Table 1:** Pore size of foam dressings

	Pore size (μm)			
	Surface		Cross-section	
	Size distribution	Average	Size distribution	Average
Medifoam®silver	25–75	69.2	100–350	274.8
Allevyn®Ag	70–90	78.6	370–500	422.4
Mepilex®Ag	100–250	150.8	350–500	441.6
Polymem®silver	130–250	195	900–1500	1236

The morphology of foam dressings in surface and cross-section were assessed with FE-SEM as shown in Fig. 2. The morphology of surface as a wound contact layer was shown in Fig. 2A. Surface of Medifoam® Silver showed uniform and small pore size as shown in Fig. 2A(a). In the surface area, Polymem®silver had a largest pore-size, followed by Mepilex®Ag, then, by Allevyn® Ag in descending order. The pore size of the cross sections was the same in order, largest in Polymem®silver, followed by Mepilex®Ag and Allevyn® Ag. The size of pore in foam dressing is summarized in Table 1.

**Fig. 3:**
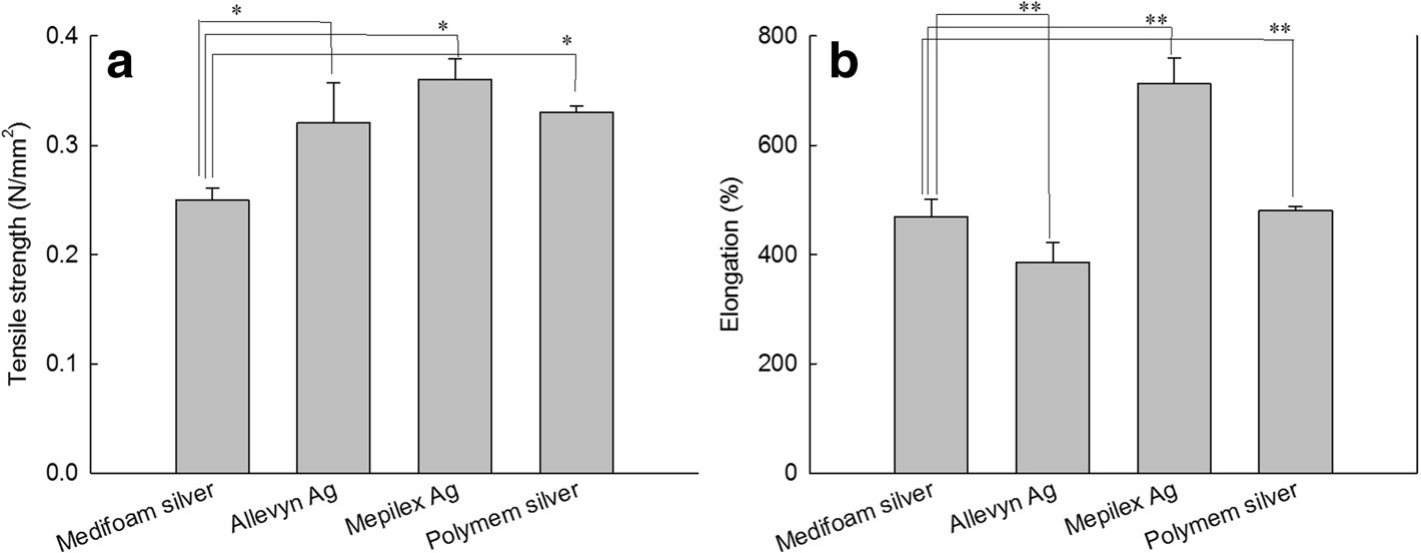
Tensile strength (**a**), and elongation (**b**) of foam dressings. *,** : *p* < 0.05

Figure 3 showed tensile strength and elongation of foam dressings. As shown in Fig. 3(a), Mepilex®Ag showed good tensile strength and elongation. Medifoam®Ag has low tensile strength but its elongation was optimal. Allevyn®Ag has good tensile strength but its elongation value was low.

### Absorption and retention properties of foam dressings

**Fig. 4:**
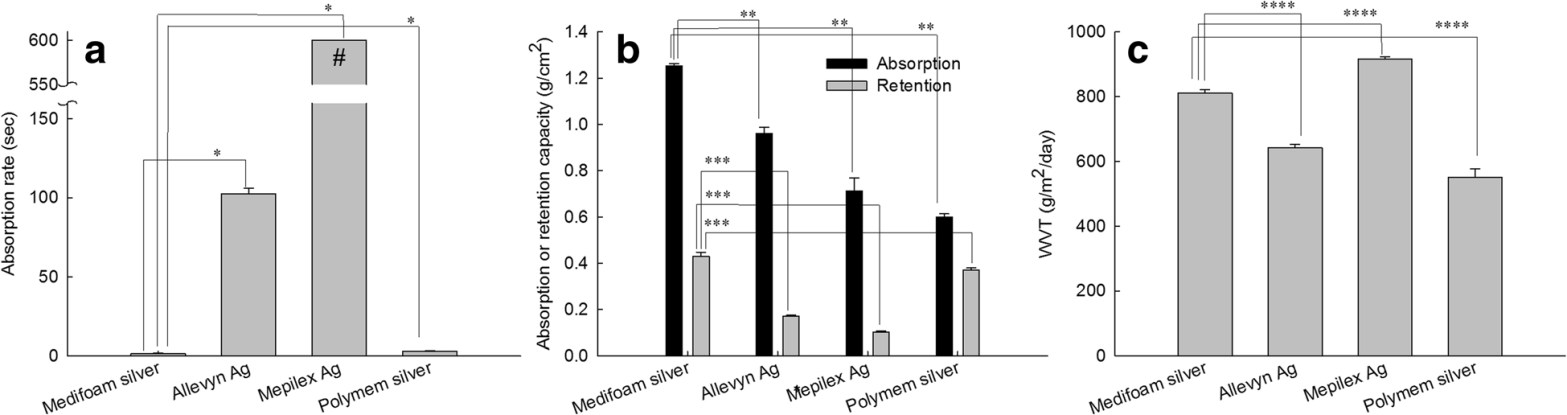
Comparison of absorption rate (**a**), absorption/retention capacity (**b**), and WVT value (**c**) of foam dressings. *: *p* < 0.001; **,***,**** : *p* < 0.01

Absorption properties of foam dressings were shown in Fig. 4. As shown in Fig. 4(a), absorption rate of Medifoam®Silver and Polymem®silver was faster than other dressings. Mepilex®Ag required the longest absorption period. Furthermore, Medifoam®Silver showed high absorption/retention capacity as shown in Fig. 4(b), i.e. the average absorption and retention capacity of Medifoam®Silver was 1.25 and 0.43 g/cm^2^, respectively.

### Antibacterial activity of silver-containing dressings

**Fig. 5:**
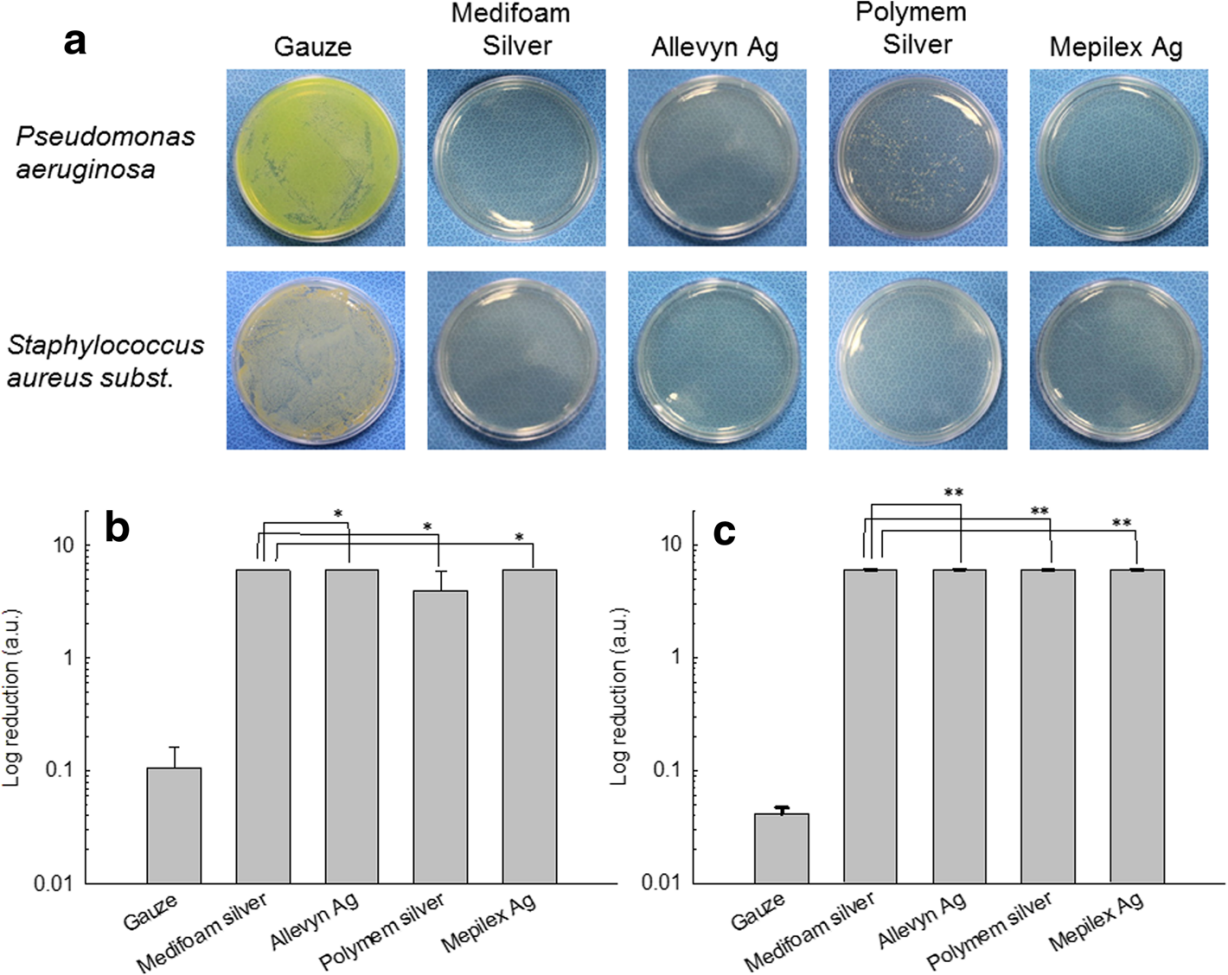
(**a**) comparison of antibacterial activity in vitro of foam dressings. Antibacterial effect of silver dressing against *Pseudomonas aeruginosa* and *Staphylococcus aureus subst*. (ATCC6538). **b** and **c** were reduction ratio of *Pseudomonas aeruginosa* and *Staphylococcus aureus subst*., respectively. Logarithmic reduction ratio was calculated as detailed in the method section (Antibacterial activity). *, ** : *p* < 0.05

As in Fig. 5(a), antibacterial activity against *Staphylococcus aureus subst*. and Psudomonas aeruginosa was clearly recognized in all silver dressing-treated samples, while gauze-treated samples showed full of microbial growth. Numerically, all silver dressing-treated samples showed 99.9 % bacterial inhibition against *Staphylococcus aureus subst*. and Psudomonas aeruginosa in comparison to gauze-treated sampled (Fig. 5(b) and (c)). These results indicated that Medifoam®Silver, has excellence bacteriocidal effect against both *Pseudomonas aeruginosa* and *Staphylococcus aureus subst*. Silver dressing including a silver sulfadiazine had good antibacterial activity in a diabetic rat model with a MRSA infection [11]. Therefore, Medifoam®Silver, which is also wound dressing containing silver sulfadiazine, can be assumed to be effective in inhibition of bacterial growth in wound healing. The data suggests Medifoam®Silver as a promising candidate for wound care.

## Discussion

Excessive exudate in wound healing management induces delayed healing process, maceration of peri-wound skin and embarrassing leakage for the patient [2]. The pore size and pore morphology of wound dressing material may contribute the potential of wound dressing in the wound healing process. We previously reported that small pore size. Pore structure and its uniformity of Medifoam® N is related to moisture-vapor transmission rate and fluid absorption/retention capacity, and these excellent physicochemical properties must be contributed to improve wound healing process [13]. The smaller pore size and uniformity of the surface and cross-section preferentially affect the microenvironment of wound area with its property of capillary absorption, retention capacity, WVT for exudate. Chitrattha and Phaechamud reported that porosity and pore size significantly influenced on WVT, drug release rate and oxygen transmission rate, and these properties were deeply related to antibacterial activity [17].

In addition, the pore size considered to play an essential role in minimizing fibroblast/keratin ingrowth into the device and reducing secondary damage upon dressing change [18]. Moreover, dressing material having wet and incubator-like microenvironment can provide clear advantages such as prevention of dehydration, control of bacterial penetration, and bullae formation [19]. Medifoam®Silver features an absorptive SSD-containing polyurethane foam layer between a semi-permeable absorptive polyurethane film wound contact layer and an outer layer of breathable film. These physical properties in thickness, density as well as pore strucuture and size were designed to optimize absorption and retention properties in clinical wound healing practice. Furthermore, the absorption and retention rates for silver dressings have been limitedly reported. One study investigated the water absorption rates of four dressings (carbon fiber, hydrogel, silver nanoparticle, and Vaseline gauze) used in patients with partial-thickness burns [20]. The absorption rates were highest with the carbon fiber dressing, with the rates of the silver nanoparticle dressing being next highest. Compared with wounds without a dressing, evaporation from dressed wounds decreases [20]. In another study comparing various wound dressings, a nanoparticle silver dressing showed good absorptive properties, absorbing up to 30 % of its weight after 72 h in a high-humidity environment [21].

The current study showed the good absorption and retention capacity of Medifoam®Silver. Advanced wound dressing material having ideal physical/structural properties can effectively absorb excess exudate from wound interface, prevent dryness of wound by maintaining balanced moisture condition, and provide barrier function against bacterial infections [2, 4]. Therefore, Medifoam®Silver would have optimal performance in absorption and retention of biological solutions and secretion from actual wounds. Furthermore, Medifoam®Silver has certain tensile strength and elongation properties as shown in Fig. 3. Those were considered to be an important factor for maintaining physicochemical properties, controlling drug release/WVT/MVTR and antibacterial activity [22, 23]. The absorption and retention capacity of Medifoam®Silver was good in comparison to those of several commercially available silver dressings. Given the detrimental effects of excessive moisture loss and potential for infection in wounds, highly absorptive and moisture retentive dressings such as BETAplast, are a welcomed addition to wound management armamentarium.

When physicochemical and structural properties of Medifoam®Silver were examined, it demonstrated suitable thickness, density, tensile strength and elongation. The morphologic study of Medifoam®Silver has shown the smallest pore size and the best uniformity of pore among tested dressings in both contact layer and cross-section. It also exhibited fast absorption rate, good absorption/retention capacity and good WVT value. In addition, antibacterial activity of Medifoam®Silver was equivalent to other commercially available silver foam dressings. With its optimal physicochemical and antibacterial activity, Medifoam®Silver is expected to contribute to improved wound healing process.

## Conclusion

Our in vitro studies give a better understanding of the results achieved with Medifoam®Silver in an uncontrolled clinical study in burn wounds [24]. Randomized clinical trials of silver wound dressings are difficult to conduct because of the multitude of potential comparators, practices and the many types of wound to treat. Nevertheless, clinical case studies or even selected clinical trials may confirm insight from this study.

## Abbreviations

AAG, Allevyn® AG; ATCC, American Type Culture Collection; BS EN, British Standard European Norm; CFU, numbers of colonies; PBS, phosphate-buffered saline; PS, Polymem® Silver; PU, polyurethane; WVT, water-vapor transmission

## References

[1] Leaper DJ, Schultz G, Carville K, Fletcher J, Swanson T, Drake R (2012). Extending the TIME concept: what have we learned in the past 10 years?(*). Int Wound J.

[2] Kotz P, Fisher J, McCluskey P, Hartwell SD, Dharma H (2009). Use of a new silver barrier dressing, ALLEVYN Ag in exuding chronic wounds. Int Wound J.

[3] Fujiwara T, Hosokawa K, Kubo T (2013). Comparative study of antibacterial effects and bacterial retentivity of wound dressings. Eplasty.

[4] Beam JW (2009). Topical silver for infected wounds. J Athl Train.

[5] Hiro ME, Pierpont YN, Ko F, Wright TE, Robson MC, Wyatt G (2012). Comparative evaluation of silver-containing antimicrobial dDressings on in vVitro and in vivo processes of wound healing. ePlasty.

[6] Castellano JJ, Shafii SM, Ko F, Donate G, Wright TE, Mannari RJ, Payne WG, Smith DJ, Robson MC (2007). Comparative evaluation of silver-containing antimicrobial dressings and drugs. Int Wound J.

[7] Palmieri TL, Greenhalgh DG (2002). Topical treatment of pediatric patients with burns: a practical guide. Am J Clin Dermatol.

[8] Paquet P, Piérard GE (2010). Topical treatment options for drug-induced toxic epidermal necrolysis (TEN). Expert Opin Pharmacother.

[9] Silver S, le Phung T, Silver G (2006). Silver as biocides in burn and wound dressings and bacterial resistance to silver compounds. J Ind Microbiol Biotechnol.

[10] Khundkar R, Malic C, Burge T (2010). Use of Acticoat dressings in burns: what is the evidence?. Burns.

[11] Lee JH, Ja Kwak J, Shin HB, Jung HW, Lee YK, Yeo ED, Seok Yang S (2013). Comparative efficacy of silver-containing dressing materials for treating MRSA-infected wounds in rats with streptozotocin-induced diabetes. Wounds.

[12] Bishop SM, Walker M, Rogers AA, Chen WY (2003). Importance of moisture balance at the wound-dressing interface. J Wound Care.

[13] Lee SM, Park IK, Kim YS, Kim HJ, Moon H, Mueller S, Jeong YI (2016). Physical, morphological, and wound healing properties of a polyurethane foam-film dressing. Biomater Res.

[14] Yunoki S, Kohta M, Ohyabu Y, Iwasaki T (2015). In vitro parallel evaluation of antibacterial activity and cytotoxicity of commercially available silver-containing wound dressings. Plast Surg Nurs.

[15] Ip M, Lui SL, Poon VK, Lung I, Burd A (2006). Antimicrobial activities of silver dressings: an in vitro comparison. J Med Microbiol.

[16] Abarca-Buis RF, Munguía NM, Gonzalez JM, Solís-Arrieta L, Osorio LS, Krötzsch E (2014). Silver from polyurethane dressing is delivered by gradient to exudate, tissue, and serum of patients undergoing negative-pressure wound treatment. Adv Skin Wound Care.

[17] Chitrattha S, Phaechamud T (2016). Porous poly(DL-lactic acid) matrix film with antimicrobial activities for wound dressing application. Mater Sci Eng C Mater Biol Appl.

[18] Levina EM, Kharitonova MA, Rovensky YA, Vasiliev JM (2001). Cytoskeletal control of fibroblast length: experiments with linear strips of substrate. J Cell Sci.

[19] Junker JP, Caterson EJ, Eriksson E (2013). The microenvironment of wound healing. J Craniofac Surg.

[20] Chen J, Han CM, Su GL, Tang ZJ, Su SJ, Lin XW (2007). Randomized controlled trial of the absorbency of four dressings and their effects on the evaporation of burn wounds. Chin Med J (Engl).

[21] Aramwit P, Muangman P, Namviriyachote N, Srichana T (2010). In vitro evaluation of the antimicrobial effectiveness and moisture binding properties of wound dressings. Int J Mol Sci.

[22] Kavoosi G, Dadfar SM, Purfard AM (2013). Mechanical, physical, antioxidant, and antimicrobial properties of gelatin films incorporated with thymol for potential use as nano wound dressing. J Food Sci.

[23] Wang X, Cheng F, Gao J, Wang L (2015). Antibacterial wound dressing from chitosan/polyethylene oxide nanofibers mats embedded with silver nanoparticles. J Biomater Appl.

[24] Ha TS, Cho YS, Kim DH, Hur J, Chun W, Kim JH, Shin HS, Kim AS, Noh SY (2007). The clinical effectiveness of medifoam-silver in burns. J Korean Burn Soc.

