# Erratum to: Superior absorption and retention properties of foam-film silver dressing versus other commercially available silver dressing

**DOI:** 10.1186/s40824-016-0085-z

**Published:** 2016-12-05

**Authors:** Seung Moon Lee, Il Kyu Park, Yong Soo Kim, Hyun Jung Kim, Hanlim Moon, Stefan Mueller, Harsha Arumugam, Young-IL Jeong

**Affiliations:** 1 Genewel Co. Ltd, Gyeonggi-do, Korea; 2 Mundipharma Pte. Ltd, Singapore, Singapore; 3 Biomedical Research Institute, Pusan National University Hospital, 179 Gudeok-ro, Seo-gu, Busan, 602-739 Republic of Korea

## Erratum

The original article contains an error [1] in the **Discussion** section, in the following sentence:

“[…] highly absorptive and moisture retentive dressings such as BETAplast, are a welcomed addition to wound management armamentarium.”

The mention of ‘BETAplast’ in this sentence should instead be ‘Medifoam® Silver’.
